# Simultaneous detection and differentiation of classical Muscovy duck reovirus and goose-origin Muscovy duck reovirus by RT-qPCR assay with high-resolution melting analysis

**DOI:** 10.3389/fvets.2024.1459898

**Published:** 2024-10-24

**Authors:** Zhuoran Xu, Hongwei Liu, Xin Zheng, Xiaoxia Cheng, Shao Wang, Guangju You, Xiaoli Zhu, Min Zheng, Hui Dong, Shifeng Xiao, Li Zeng, Xiancheng Zeng, Shaoying Chen, Shilong Chen

**Affiliations:** ^1^Institute of Animal Husbandry and Veterinary Medicine, Fujian Academy of Agricultural Sciences, Fuzhou, China; ^2^College of Animal Sciences, Fujian Agriculture and Forestry University, Fuzhou, China; ^3^Fujian Animal Diseases Control Technology Development Center, Fuzhou, China

**Keywords:** classical Muscovy duck reovirus, goose-origin Muscovy duck reovirus, duplex real-time RT-PCR, high-resolution melting, differential diagnosis

## Abstract

**Introduction:**

Classical Muscovy duck reovirus (C-MDRV) and goose-origin Muscovy duck reovirus (Go-MDRV) infections cause “Liver white-spots disease” in Muscovy duckling and gosling. It is difficult to differentiate the infections caused by C-MDRV and Go-MDRV using conventional serological methods.

**Methods:**

Specific primers were designed and synthesized according to σNS and λA nucleotide sequences of C-MDRV and Go-MDRV, respectively. The PCR amplified products were cloned into the pMD-18-T vector. The recombinant plasmid DNA was used to establish an SYBR Green І based duplex real-time PCR assay for the simultaneous detection and differentiation of C-MDRV and Go-MDRV using high-resolution melting (HRM) analysis. The specificity, sensitivity, and repeatability of the methodology were examined based on the optimization of the reaction system and amplification conditions.

**Results:**

C-MDRV and Go-MDRV were identified by their distinctive melting temperatures with 84.50 ± 0.25°C for C-MDRV and 87.50 ± 0.20°C for Go-MDRV, respectively. The amplifications were specific, and other non-targeted waterfowl viruses employed in this study did not show normalized melting peaks. The intra- and inter-assay coefficients of variations were between 0.05 and 1.83%, demonstrating good repeatability. The detection limits of this assay were 51.4 copies·μl^−1^ for C-MDRV and 61.8 copies·μl^−1^ for Go-MDRV, respectively. A total of 45 clinical samples were tested by RT-qPCR, with positive rates of 15.56% for C-MDRV and 22.22% for Go-MDRV, without co-infections.

**Discussion:**

These results suggest that this duplex RT-qPCR method is highly sensitive, specific, and reproducible. The HRM assay established in this study provides a powerful tool for the differential detection and epidemiological investigation of C-MDRV and Go-MDRV.

## 1 Introduction

Reovirus infection in waterfowl has caused substantial economic losses in global waterfowl production, particularly affecting Muscovy ducks. Classical Muscovy duck reovirus (C-MDRV) infection was first reported in Muscovy ducklings in South Africa in 1950 (1), and the C-MDRV strain 89026 was initially isolated in 1972 (2). The disease emerged in Chinese Muscovy duck flocks in the early 1990s (3). C-MDRV infection can result in a range of clinical symptoms, including weakness, arthritis (lameness), watery diarrhea, and stunted growth. The typical pathological manifestation is numerous white pin-head necrotic foci distributed in the liver and spleen (4). Thus, the disease is commonly known as “Liver white spots disease” in Muscovy duck (5).

Reovirus infection in goslings (GRV infection) was first reported in Hungary in the 1990s and was characterized by splenitis and hepatitis with miliary white necrotic foci during the acute phase (6, 7). GRV infection has also been reported in China since 2002, with clinical symptoms similar to GRV infection in Hungary and C-MDRV infection, including weakness, locomotor disorders, arthritis, and diarrhea (8, 9). Based on similar genomic characteristics, GRV and C-MDRV are recommended to be classified as specified genogroup of avian reoviruses (8). Since 2020, GRV infection has occurred frequently in Chinese goose breeding areas, presenting typical characteristics such as numerous white necrotic foci in the liver and spleen (10, 11). The pathogenic agent was identified as a novel GRV (N-GRV) strain with natural recombination from different waterfowl reoviruses (10, 11). It has been proposed to name these GRV strains goose-origin Muscovy duck reovirus (Go-MDRV) (12).

Waterfowl reoviruses are nonenveloped, icosahedral viruses. Their genomes consist of 10 segments of double-stranded RNA (dsRNA), which can be divided into small genome fragments (S1, S2, S3, S4), medium genome fragments (M1, M2, M3), and large genome fragments (L1, L2, L3) based on their electrophoretic mobility (6, 13). C-MDRV and Go-MDRV belong to the genus *Orthoreovirus* of the reovirus family, causing similar clinical symptoms (14–16) and exhibiting a high genomic homology of 89.5–98.5% (10). Differentiating between them using conventional serological methods is challenging (17). The present methods for detecting C-MDRV or Go-MDRV include enzyme-linked immunosorbent assay (ELISA) (18), immunofluorescence assay (IFA) (19), reverse transcription-polymerase chain reaction (RT-PCR) assay (12, 20, 21), semi-nested RT-PCR (22), *Taq*Man-based real-time RT-PCR assay (23, 24), and SYBR Green I based real-time RT-PCR assay (25). However, these methods could not be used for simultaneous detection and differentiation of C-MDRV and Go-MDRV. C-MDRV and Go-MDRV are pathogenic to Muscovy ducklings and goslings, causing similar symptoms, which makes it difficult to determine whether the “Liver white spots disease” is caused by C-MDRV or Go-MDRV infection. It is urgent to establish a method for rapid detection and differentiation of C-MDRV or Go-MDRV.

Compared with the traditional RT-PCR and serological detection methods, real-time PCR assay has the advantages of quantitative, rapid, accurate, and high sensitivity. Compared with *Taq*Man-based real-time PCR, SYBR green I based real-time PCR has benefits including low cost, simplicity, and exclusion of non-specificity by melting curve analysis (26). In this study, an SYBR Green I based duplex RT-qPCR assay was developed to detect and distinguish C-MDRV and Go-MDRV based on their different melting temperatures.

## 2 Materials and methods

### 2.1 Viruses and clinical samples

The Go-MDRV strain JS2022, C-MDRV strain MW9710, and control viral strains were provided by the Laboratory of Animal Virology of the Institute of Animal Husbandry and Veterinary Medicine, Fujian Academy of Agriculture Sciences, China. The control strains included goose parvovirus (GPV), Muscovy duck parvovirus (MDPV), duck aviadenovirus serotype B2 (DAdV-B2), duck Tembusu virus (DTMUV), duck enteritis virus (DEV), novel duck reovirus (NDRV), duck hepatitis A virus type 1 (DHAV-1), and duck paramyxovirus (DPMV).

A total of 45 clinical “Liver white spots disease” samples of dead Muscovy ducklings and goslings were collected from different waterfowl farms in the south of China between 2020 and 2023. The samples were stored at −20°C and examined simultaneously. Mixed homogenates of heart, liver and spleen (30% w/v) were prepared for viral DNA and RNA extraction according to the method described previously (27). Viral DNA/RNA was extracted with the FastPure Viral DNA/RNA Mini Kit (Vazyme Biotech, Nanjing, China) according to the manufacturer’s instructions. Viral RNA was reverse-transcribed to obtain complementary DNA (cDNA) using HiScript®II first strand cDNA synthesis kit (Vazyme Biotech, Nanjing, China) according to the manufacturer’s instructions. The cDNA and viral DNA were used immediately for qPCR or stored at −80°C for future use.

### 2.2 Primer design

Among the various strains of the avian orthoreoviruses, the genes with great differences in nucleotide sequence homology are the target genes for primer designing. The nucleotide sequences of the C-MDRV σNS gene (Accession no: KC508655, KF306090, KJ569582, DQ066923, DQ325536) and the nucleotide sequences of Go-MDRV λA gene (Accession no: MZ546418, OP598202, OK626883, OR890071) were aligned by Clustal W using the MegAlign program (DNASTAR Inc., Madison, WI, USA). qPCR primers targeting the C-MDRV σNS gene and another pair of primers targeting the Go-MDRV λA gene were designed using Oligo 6 software (Med. Probe, Oslo, Norway; Table 1). The primer sequences were aligned to confirm the possibility of cross-reactivity with other members of the avian orthoreoviruses. The primers were synthesized by Tsingke Biotechnology Beijing Co., Ltd., Beijing, China.

**Table 1:** Primers designed and used in the duplex RT-qPCR assay for the simultaneous detection of C-MDRV and Go-MDRV.

Primes	Sequence (5′ → 3′)	Target gene	Primer location (nt)	Product size (bp)
C-MDRV-F	ACATCCTGACTCGCGATTTA	σNS	250-269^a^	126
C-MDRV-R	CACCATAAACTTGAGCCACA		356-375^a^	
Go-MDRV-F	TGAAGTCCGACAACCCTACC	λA	125-144^b^	324
Go-MDRV-R	CGTCATTGTCCACGGATCCA		429-448^b^	

a Oligonucleotide position about C-MDRV strain ZJ2000M segment σNS sequence (GenBank accession no. KF306090).

b Oligonucleotide position about Go-MDRV isolate JS2022 segment λA sequence (GenBank accession no. OP598202).

### 2.3 Preparation of standard plasmids

The PCR was performed using the primers described in Table 1 to amplify the σNS and λA DNA fragments. The reaction volume was 20 μl, containing 10 μl of 2 × *Taq* Master Mix (Dye Plus; Vazyme Biotech, Nanjing, China), 2 μl of cDNA template, 1 μl of each primer (10 μM), and 6 μl of ddH_2_O. The cycling protocol was as follows: initial denaturation at 95°C for 3 min; 35 cycles of denaturation at 95°C for 15 s, annealing at 58°C for 15 s, and extension at 72°C for 30 s, and final extension at 72°C for 5 min. The PCR products were purified and cloned into the pMD18-T Vector to obtain the recombinant plasmids pMD18-C-MDRV and pMD18-Go-MDRV. The DNA of recombinant plasmids was verified by sequencing. The concentration and purity of the two standard plasmids were quantified using a DS-11 Spectrophotometer (DeNovix, Wilmington, DE, USA). The copy numbers of each cloned gene were calculated according to the method described previously (28). Each recombinant standard plasmid was diluted 10-fold in TE buffer and used to construct the standard curves.

### 2.4 PCR-HRM assay

C-MDRV and Go-MDRV were detected by single RT-qPCR in a 20 μl reaction mixtures containing 10 μl of PerfectStart® Green qPCR SuperMix (TransGen Biotech, Beijing, China), 0.5 μl each of 10 μM forward and reverse primers of C-MDRV or Go-MDRV, and 1 μl of the plasmid DNA template. In the duplex qPCR, 25 μl reaction mixture comprised 12.5 μl PerfectStart® Green qPCR SuperMix, 0.4 μl each of the C-MDRV primers mix, 0.4 μl each of the Go-MDRV primers mix, 1 μl each of the plasmid templates of pMD18-C-MDRV and pMD18-Go-MDRV, and RNase-free H_2_O was added to a total volume of 25 μl. Amplification was performed on a LightCycler® 96 Instrument (Roche, Basel, Switzerland) using the following cycling program: initial denaturation at 94°C for 60 s, followed by 40 cycles of denaturation at 94°C for 5 s, annealing at 60°C for 15 s, and extension at 72°C for 6 s. Fluorescence signals were collected at the end of each cycle. HRM software (Roche, Basel, Switzerland) was used to analyze the data.

### 2.5 Establishment of standard curves

The 10-fold serial dilutions of the standard plasmid DNA for the C-MDRV σNS gene or Go-MDRV λA gene were used in the qPCR amplification. The standard curves were constructed by plotting the Cycle Threshold (Ct) values on the y-axis and logarithmic starting concentrations along the x-axis.

### 2.6 Sensitivity, specificity, and reproducibility evaluations

The recombinant plasmid DNA of the C-MDRV σNS gene or Go-MDRV λA gene was serially diluted 10-fold from 10^8^ copies·μl^−1^ to 10^0^ copies·μl^−1^. Seven concentrations (10^8^ to 10^2^ copies·μl^−1^) of the standard C-MDRV and Go-MDRV plasmids were detected by this duplex qPCR assay to assess the minimum detection limits. The intra- and inter-group repeatability of seven concentrations of standard C-MDRV and Go-MDRV plasmids was determined using this duplex qPCR assay detection by calculating the coefficients of variation (CVs). The DNA or cDNA of seven control virus strains (GPV, MDPV, DAdV-B2, DTMUV, DEV, NDRV, DHAV-1, and DPMV) and RNase-free H_2_O were used as the negative control to evaluate the specificity of this assay.

### 2.7 Evaluation of clinical samples by real-time PCR and conventional PCR

A total of 45 clinical samples from diseased Muscovy ducklings and goslings from different waterfowl farms were detected by the established duplex RT-qPCR, single RT-qPCR, and the conventional RT-PCR assay (12). The positive samples were selected for sequencing and the National Center for Biotechnology Information (NCBI) Basic Local Alignment Search Tool (BLAST) analysis to confirm the reliability of the assay. The positive detection rates of the three assays were compared.

## 3 Results

### 3.1 Validation of the RT-qPCR-HRM assay

**Figure 1:**
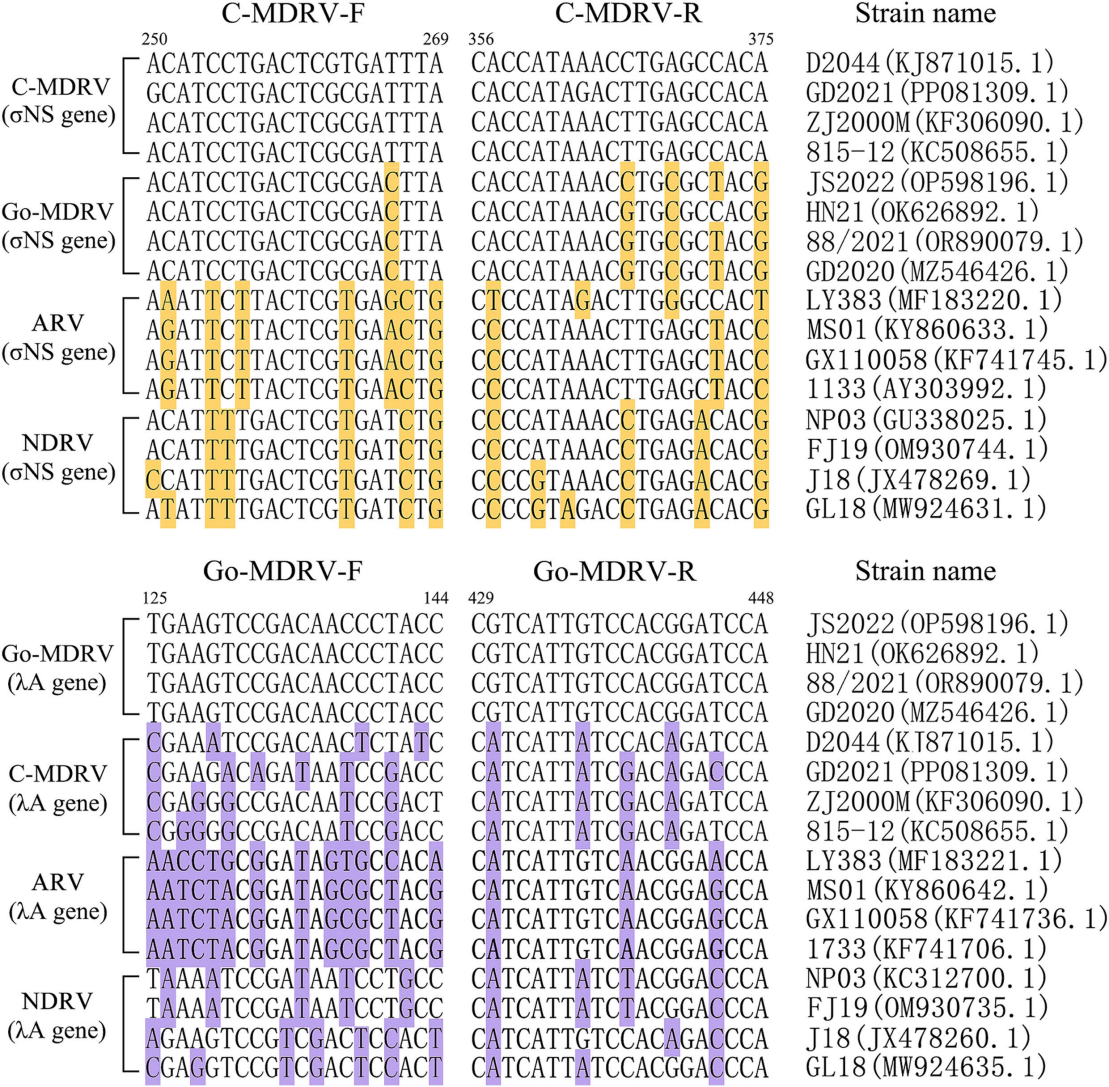
Nucleotide alignment of primer sequences from different members of avian orthoreoviruses. GenBank accession numbers are indicated for each strainused in creating the alignment. The bases that differ from primer sequences are highlighted by background color.

**Figure 2:**
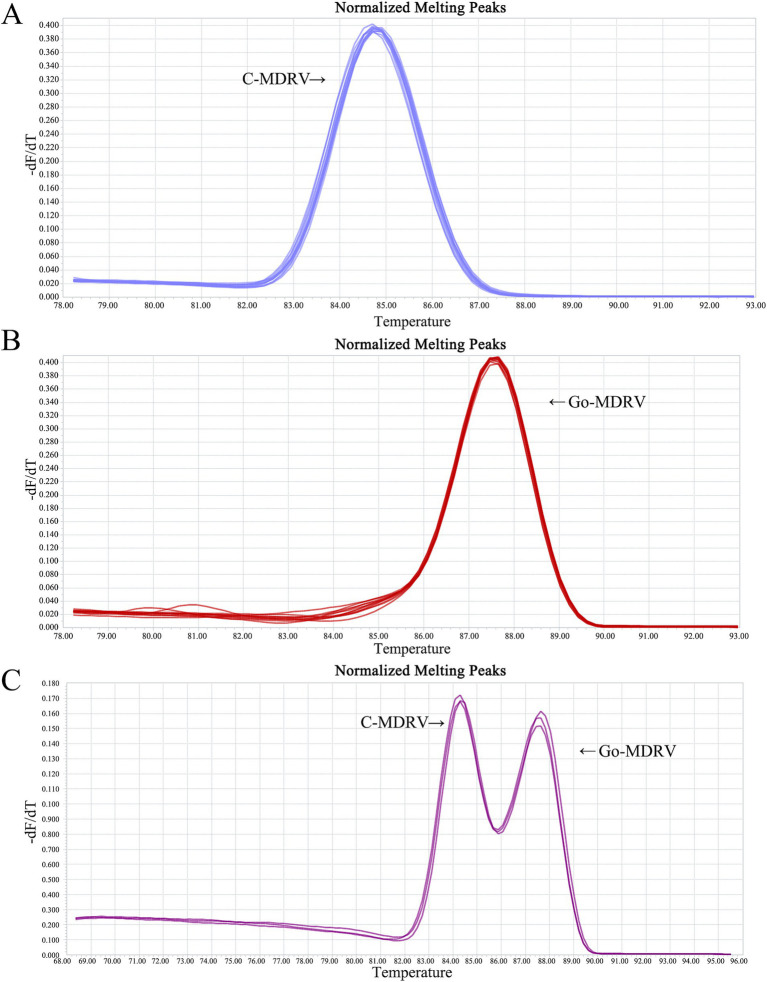
Melting curve analysis of C-MDRV and Go-MDRV. **(A)** Melting curve analysis of C-MDRV singleplex RT-qPCR, with a *T_m_* value of 84.50°C. **(B)** Melting curve analysis of Go-MDRV singleplex RT-qPCR, with a *T_m_* value of 87.50°C. **(C)** Duplex normalized melting curve analysis for C-MDRV and Go-MDRV, showing *T_m_* values of 84.50 and 87.50°C, respectively, consistent with the results of the singleplex melting curve analyses.

The result of primer sequence alignment was shown in Figure 1. The primer sequences designed in this study only matched the C-MDRV σNS gene or Go-MDRV λA gene sequences, respectively, suggesting the amplification had a good specificity. As shown in Figures 2A,B, positive fluorescent signals for C-MDRV and Go-MDRV were obtained with the respective single RT-qPCR assays with the melting temperature (*T_m_*) of 84.50°C and 87.50°C, respectively. It was sufficient to differentiate C-MDRV and Go-MDRV by the normalized melting curves with double peaks (Figure 2C). The mean and standard deviation of *Tm* of C-MDRV and Go-MDRV were 84.50 ± 0.25°C and 87.50 ± 0.20°C, respectively. Only double-specific peaks indicated no primer dimers and cross-reactivity between primer sets.

### 3.2 Standard curves of C-MDRV and Go-MDRV

**Figure 3:**
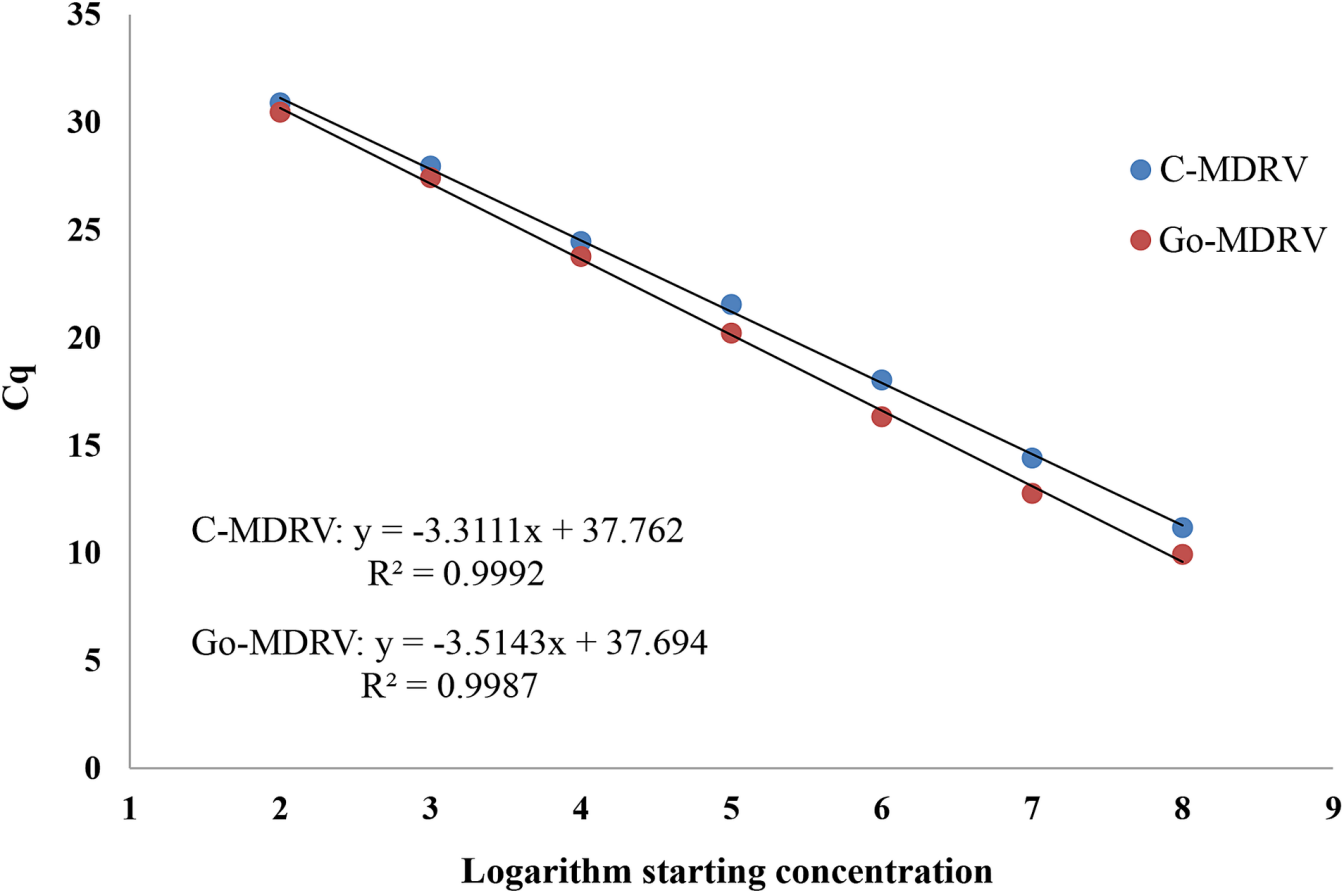
Standard curve analysis of the standard plasmid dilutions by SYBR Green І based real-time RT-PCR. The standard curve was constructed with concentrations ranging from 5.14 × 10^2^ to 5.14 × 10^8^ copies·μl^−1^ for C-MDRV and 6.18 × 10^2^ to 6.18 × 10^8^ copies·μl^−1^ for Go-MDRV. The standard curve of C-MDRV is y = −3.3111x + 37.762, with an R^2^ value of 0.9992. The Go-MDRV standard curve is y = −3.5143x + 37.694, with an R^2^ value of 0.9987.

The standard plasmids ranging from 5.14 × 10^2^ to 5.14 × 10^8^ copies·μl^−1^ for C-MDRV and 6.18 × 10^2^ to 6.18 × 10^8^ copies·μl^−1^ for Go-MDRV were used to construct the standard curves. The standard curve equations for C-MDRV and Go-MDRV were y = −3.3111x + 37.762 and y = −3.5143x + 37.694, respectively (Figure 3). The amplification efficiencies were 100.45 and 92.55% for C-MDRV and Go-MDRV, respectively. Additionally, the R^2^ values of C-MDRV and Go-MDRV were 0.9992 and 0.9987, respectively.

### 3.3 Sensitivity, specificity, and reproducibility analysis

**Figure 4:**
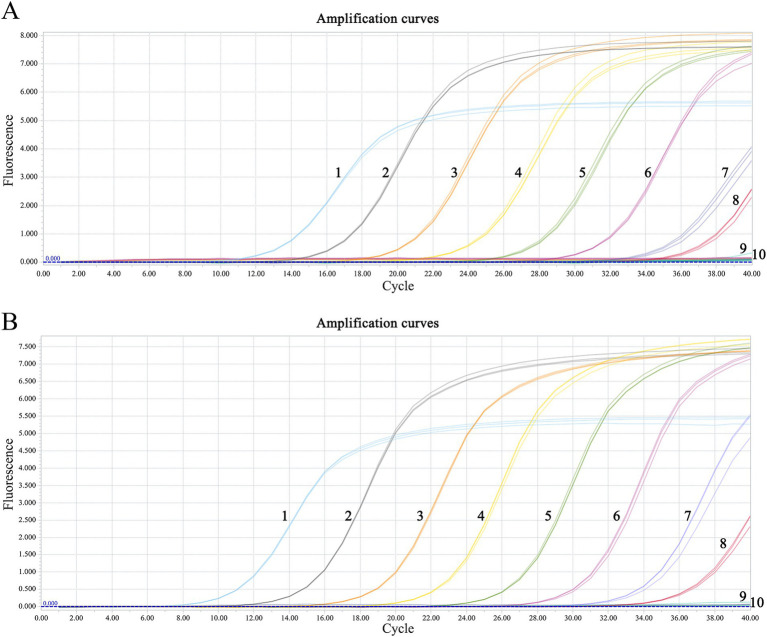
Sensitivity analysis of duplex RT-qPCR assay for detecting C-MDRV and Go-MDRV. **(A)** The amplification curve of C-MDRV, with the lowest detection limit of 51.4 copies·μl^−1^; NO. 1–9 represent 5.14 × 10^8^ ~ 5.14 × 10^0^ copies·μl^−1^, respectively; NO. 10 represents negative control. **(B)** The amplification curve of Go-MDRV, with the lowest detection limit of 61.8 copies·μl^−1^; NO. 1–9 represent 6.18 × 10^8^ ~ 6.18 × 10^0^ copies·μl^−1^, respectively; NO. 10 represents negative control.

**Figure 5:**
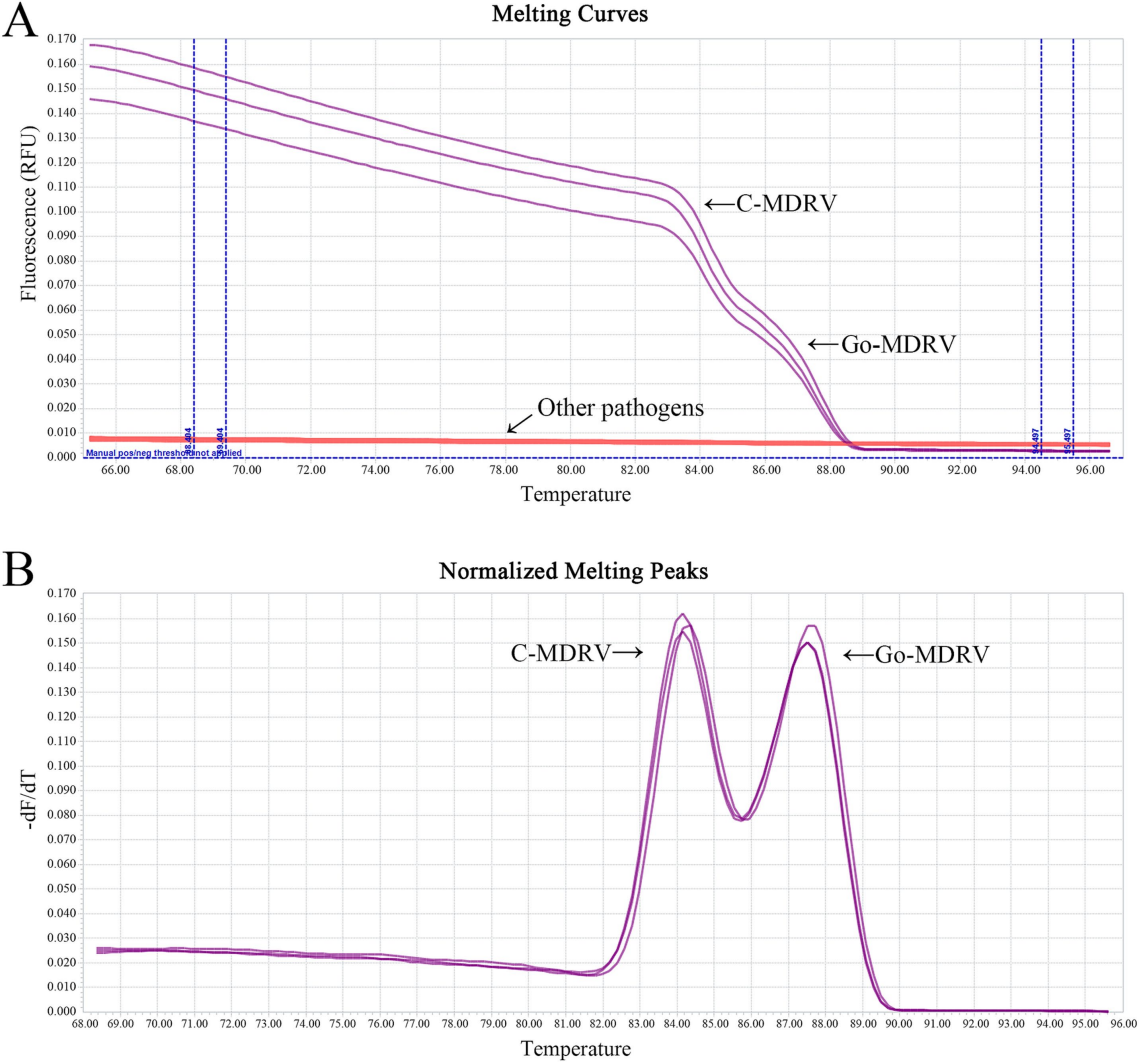
Melting curve analysis **(A)** and normalized melting peak analysis **(B)** for evaluating the specificity of the duplex SYBR Green I based real-time RT-PCR assay. No cross-reactions were detected with GPV, MDPV, DAdV-B2, DTMUV, DEV, NDRV, DHAV, and DPMV, or the negative control.

The minimum detection limits were 51.4 copies·μl^−1^ for C-MDRV and 61.8 copies·μl^−1^ for Go-MDRV (Figure 4). The RT-qPCR-HRM assay demonstrated highly specific. There were no specific normalized melting peaks for GPV, MDPV, DAdV-B2, DTMUV, DEV, NDRV, DHAV, or DPMV (Figure 5). The intra- and inter-assay coefficients of variation for C-MDRV, determined from three parallel tests, ranged from 0.05 to 0.57% and 0.23 to 0.97%, respectively. For Go-MDRV, the intra- and inter-assay coefficients of variation were 0.07 to 0.37% and 0.40 to 1.83%, respectively (Table 2). The data from the intra- and inter-assay reproducibility tests indicated that this RT-qPCR-HRM assay was reproducible.

**Table 2:** Intra- and inter-assay coefficients of variation for the duplex SYBR Green I based real-time RT-PCR assay of C-MDRV and Go-MDRV.

	DNA standard (copies·μl^−1^)	The Ct values of intra-assay		The Ct values of inter-assay	
		Means ± SD	CV%	Means ± SD	CV%
C-MDRV	5.14 × 10^8^	11.19 ± 0.03	0.27	11.25 ± 0.07	0.64
	5.14 × 10^7^	14.40 ± 0.03	0.20	14.51 ± 0.10	0.68
	5.14 × 10^6^	18.03 ± 0.01	0.05	17.96 ± 0.13	0.74
	5.14 × 10^5^	21.54 ± 0.04	0.19	21.55 ± 0.05	0.23
	5.14 × 10^4^	24.46 ± 0.06	0.26	24.62 ± 0.24	0.97
	5.14 × 10^3^	27.96 ± 0.06	0.22	27.96 ± 0.10	0.34
	5.14 × 10^2^	30.90 ± 0.17	0.57	30.90 ± 0.14	0.44
Go-MDRV	6.18 × 10^8^	9.93 ± 0.03	0.30	9.86 ± 0.07	0.71
	6.18 × 10^7^	12.73 ± 0.017	0.14	12.94 ± 0.22	1.70
	6.18 × 10^6^	16.31 ± 0.02	0.13	16.59 ± 0.30	1.83
	6.18 × 10^5^	20.16 ± 0.07	0.37	20.13 ± 0.08	0.40
	6.18 × 10^4^	23.78 ± 0.09	0.37	23.97 ± 0.22	0.92
	6.18 × 10^3^	27.37 ± 0.05	0.18	27.27 ± 0.13	0.49
	6.18 × 10^2^	30.58 ± 0.02	0.07	30.40 ± 0.12	0.40

### 3.4 Detection of clinical samples

To compare and evaluate the developed RT-qPCR and conventional RT-PCR, a total of 45 clinical samples were tested. The positive rates of C-MDRV and Go-MDRV were 15.56% (7/45) and 22.22% (10/45), respectively, with no cases of co-infection (0/45). These results were consistent with those of single RT-qPCR. The positive rates of C-MDRV and Go-MDRV detected by conventional RT-PCR were 13.33% (6/45) and 20.00% (9/45), respectively, which were lower than those obtained using the method established in this study. In addition, universal primers were used in the conventional RT-PCR assay to detect C-MDRV and Go-MDRV and were not suitable for distinguishing.

## 4 Discussion

C-MDRV and Go-MDRV infections can cause hepatic and splenic necrosis with metabolism disorders and immunosuppression. Damage to the intestinal mucosa results in enteric dysbacteriosis and the proliferation of opportunistic pathogens (29, 30). These infections are associated with an increased probability of co-infection, with fibrinous pericarditis and perihepatitis observed in the later stages of the disease (31). Some recovered birds are stunted growth, leading to substantial economic losses in the waterfowl breeding industries (32). The C-MDRV and Go-MDRV viruses share high homology in their nucleotide sequences, virus particles structural and diameter, physicochemical, biological characteristics, and molecular properties, making them difficult to distinguish using conventional serological methods (10, 33). Waterfowl reoviruses exhibit frequent natural recombination, enabling them to spread and adapt to new hosts, which results in complex phylogenetic relationships between waterfowl reoviruses (11, 34, 35). Thus, it is important to provide an accurate, rapid, and cost-effective diagnostic method for C-MDRV and Go-MDRV.

Some methods have been developed to detect C-MDRV or Go-MDRV infection, including ELISA (18), IFA (19), RT-PCR (12, 20, 21), semi-nested RT-PCR (22), and *Taq*Man-based real-time RT-PCR (23, 24). Compared with conventional RT-PCR, RT-qPCR calculates the copy numbers of viral cDNA and is simpler, faster, and more sensitive. Compared with the *Taq*Man-based real-time RT-PCR, SYBR Green I based real-time RT-PCR is cheaper and simpler. In this study, a duplex SYBR Green I based real-time RT-PCR assay was developed to amplify the σNS gene of C-MDRV and the λA gene of Go-MDRV. Compared to individual RT-qPCR assays for these viruses, the duplex assay offers the same sensitivity but with a shorter test time and broader applicability. Due to interference and competition between primer pairs, an appropriate ratio of primer concentrations was vital for developing the duplex SYBR Green I real-time RT-PCR assay.

Incorrect primer pairs can produce primer dimers, which in turn cause non-specific normalized melting peaks and sub-optimal amplification of targets. The *Tm* values of PCR products are influenced by the guanine-cytosine (GC) content and product length (36). In this study, two different gene fragments were selected for primer design, and a SYBR Green І based duplex real-time RT-PCR assay was developed for the detection and differentiation of C-MDRV and Go-MDRV by HRM analysis. The main reason for this choice is the high homology between C-MDRV and Go-MDRV, as well as the similar GC content of the two genomic fragments. Only increasing the length of PCR products could not change the *Tm* value. Choosing the same gene to design primers could lead to mutual interference, cross-reaction, and non-specific melting peaks. We used one pair of primers targeting the σNS gene or λA gene for the possibility of differentiation of C-MDRV and Go-MDRV by HRM analysis. As shown in Supplementary data 1, the *Tm* values of these two amplicons were identical, making it difficult to differentiate C-MDRV and Go-MDRV by HRM analysis. After a series of primer pair screenings and optimizations, the highly conserved regions between the σNS gene of C-MDRV and the λA gene of Go-MDRV were chosen as the sites for our primer design.

The duplex RT-qPCR assay developed here can distinguish between C-MDRV and Go-MDRV based on their different *Tm* values, which were 84.50 ± 0.25°C for C-MDRV and 87.50 ± 0.20°C for Go-MDRV. The minimum detected concentrations of C-MDRV and Go-MDRV plasmids were 51.4 and 61.8 copies·μl^−1^, respectively, which showed higher sensitivity than conventional RT-PCR (21). The results of intra- and inter-assay indicated that this method has good repeatability. This assay has no cross-reaction with other waterfowl viruses, such as GPV, MDPV, DAdV-B2, and DTMUV. A total of 45 clinical samples were identified by this duplex assay, of which 7 samples were positive for C-MDRV and 10 samples were positive for Go-MDRV, with no co-infection samples. To make clinical detection more convenient and faster, we selected 5 positive and 5 negative samples and utilized a one-step RT-qPCR assay to determine the concordance rate. As shown in Supplementary data 2, the coincidence rate between one-step RT-qPCR and duplex RT-qPCR assay was 100%. The *Tm* values of one-step RT-qPCR assay were 82.50 ± 0.20°C for C-MDRV and 85.00 ± 0.10°C for Go-MDRV. These results indicate that our designed primers can be used in a one-step RT-qPCR assay for HRM differential detection. The differences in *Tm* value between one-step and two-step RT-qPCR assays are closely related to the reagent composition (37). The duplex RT-qPCR shows a higher positivity rate than conventional RT-PCR methods. All the positive results were confirmed by sequencing, validating the stability and reliability of the duplex real-time SYBR Green I based RT-PCR method. The duplex RT-qPCR assay developed in this study facilitates the early diagnosis and surveillance of C-MDRV and Go-MDRV infections in waterfowl.

## 5 Conclusion

A duplex SYBR Green I based real-time RT-PCR assay was successfully developed in this study to distinguish C-MDRV and Go-MDRV infections. This assay is rapid, sensitive, specific, and inexpensive, making it suitable for the differential diagnosis of C-MDRV and Go-MDRV infections in clinical cases. This assay will also aid in epidemiological investigations to control the spread of C-MDRV and Go-MDRV.

## Data Availability

The datasets presented in this study can be found in online repositories. The names of the repository/repositories and accession number(s) can be found in the article/Supplementary material.
