# Supplementary material for: Simultaneous detection and differentiation of classical Muscovy duck reovirus and goose-origin Muscovy duck reovirus by RT-qPCR assay with high-resolution melting analysis
Source: Front Vet Sci. 2024 Oct 24;11:1459898. doi: 10.3389/fvets.2024.1459898 (PMC11541953; doi:10.3389/fvets.2024.1459898)
Supplement: Supplementary file 1 [file Table_1.docx]

**Supplementary data 1**

**1. Materials and methods**

**1.1 Primer design**

Based on highly conserved sequences, two pairs of qPCR primers, one for the σNS gene of C-MDRV and one for the λA gene of Go-MDRV were designed, and we designed corresponding amplification primers at the same position (Table S1). The primers were synthesized by Tsingke Biotechnology Beijing Co., Ltd China.

**1.2 RT-qPCR detection C-MDRV and Go-MDRV**

Singleplex RT-qPCR was used to detect C-MDRV and Go-MDRV individually. The 20 μL reaction mixtures contained 10 μL of PerfectStart^®^ Green qPCR SuperMix, 0.5 μL each of 10 μM forward and reverse primers, 1 μL of the cDNA template, and the final volume was adjusted to 20 μL using RNase-free H_2_O. Amplification was performed on a LightCycler^®^ 96 Instrument using the following cycling program: 94℃ for 30 sec, followed by 40 cycles of 94℃ for 5 s, 60℃ for 15 s and 72℃ for 6 s.

**2. Result**

The results indicated that the *Tm* values of the two viruses were essentially identical when using the same gene-specific primer, which makes it challenging to effectively differentiate C-MDRV and Go-MDRV with HRM analysis (Figure S1).

**TABLE S1** Primers designed and used in the RT-qPCR for detection of C-MDRV and Go-MDRV.

| Primes | Sequence (5’→3’) | Target gene | Primer location (nt) | Product size (bp) |
| --- | --- | --- | --- | --- |
| C-MDRV-F | ACATCCTGACTCGCGATTTA | σNS | 250~269^a^ | 126 |
| C-MDRV-R | CACCATAAACTTGAGCCACA |  | 356~375^a^ |  |
| Go-MDRV-F | TGAAGTCCGACAACCCTACC | λA | 125~144^b^ | 324 |
| Go-MDRV-R | CGTCATTGTCCACGGATCCA |  | 429~448^b^ |  |
| C-MDRV-F2 | CGAGGGCCGACAATCCGACT | λA | 125~144^c^ | 324 |
| C-MDRV-R2 | CATCATTATCGACAGATCCA |  | 429~448^c^ |  |
| Go-MDRV-F2 | ACATCCTGACTCGCGACTTA | σNS | 250~269^d^ | 126 |
| Go-MDRV-R2 | CACCATAAACCTGCGCTACG |  | 356~375^d^ |  |

^a^ Oligonucleotide position in reference to C-MDRV strain ZJ2000M segment σNS sequence (GenBank accession no. KF306090.1);

^b^ Oligonucleotide position in reference to Go-MDRV isolate JS2022 segment λA sequence (GenBank accession no. OP598202.1);

^c^ Oligonucleotide position in reference to C-MDRV strain ZJ2000M segment λA sequence (GenBank accession no. KF306082.1);

^d^ Oligonucleotide position in reference to Go-MDRV isolate JS2022 segment σNS sequence (GenBank accession no. OP598196.1).

**
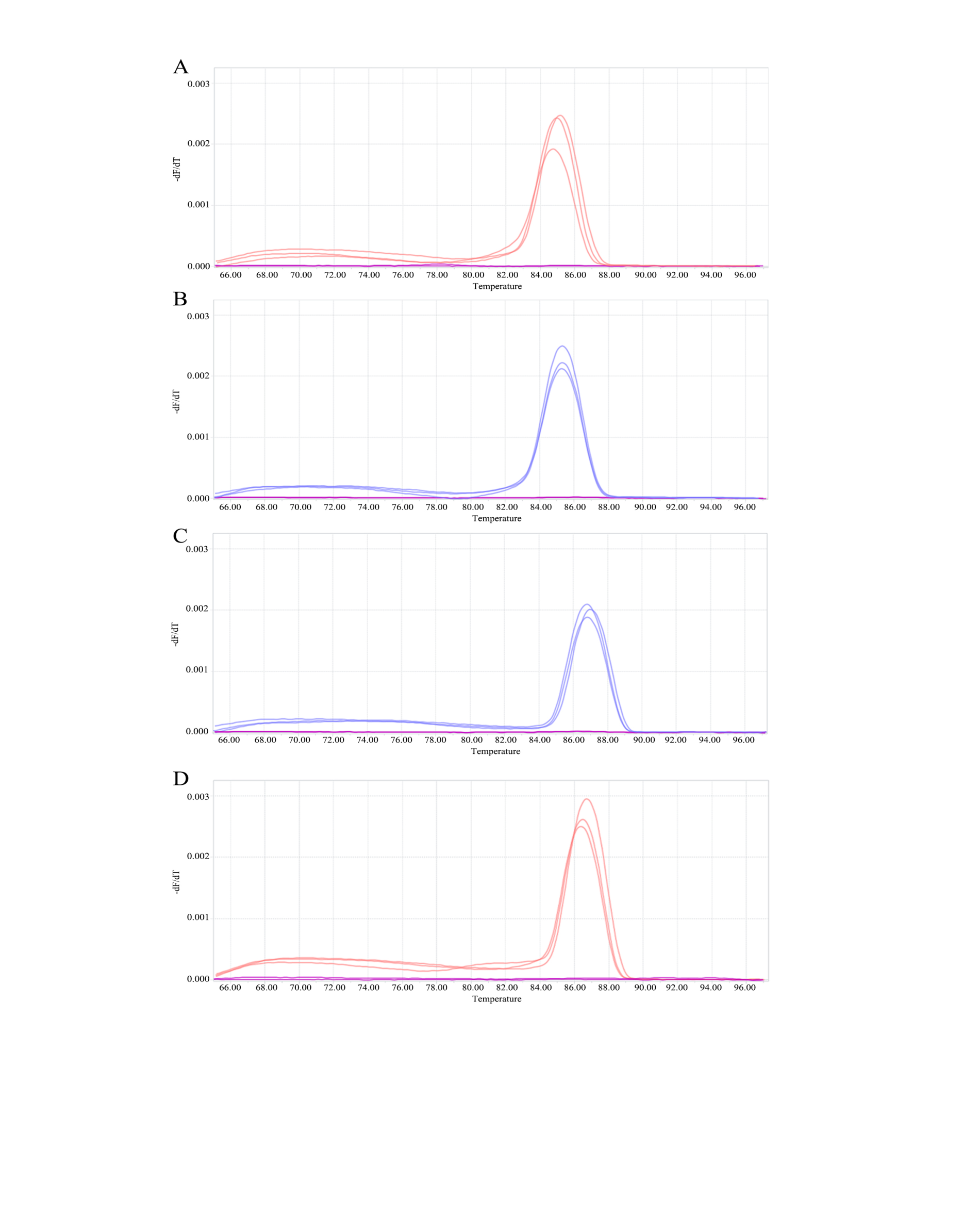
FIGURE S1** The *Tm* values of C-MDRV and Go-MDRV were essentially identical when using the same gene-specific primer. (A) Melting curve analysis of C-MDRV σNS gene singleplex RT-qPCR with a *T_m_* value of 84.5℃. (B) Melting curve analysis of Go-MDRV σNS gene singleplex RT-qPCR with a *T_m_* value of 84.5℃. (C) Melting curve analysis of C-MDRV λA gene singleplex RT-qPCR with a *T_m_* value of 87.5℃. (D) Melting curve analysis of Go-MDRV λA gene singleplex RT-qPCR with a *T_m_* value of 87.5℃.
