# Supplementary material for: Simultaneous detection and differentiation of classical Muscovy duck reovirus and goose-origin Muscovy duck reovirus by RT-qPCR assay with high-resolution melting analysis
Source: Front Vet Sci. 2024 Oct 24;11:1459898. doi: 10.3389/fvets.2024.1459898 (PMC11541953; doi:10.3389/fvets.2024.1459898)
Supplement: Supplementary file 2 [file Table_2.docx]

**Supplementary data 2**

**1. Materials and methods**

**1.1. One step RT-qPCR-HRM detection C-MDRV and Go-MDRV**

In order to make clinical detection more conveniently and fastly, we selected 5 positive and negative samples respectively and utilized one-step qPCR method to determine the coincidence rate. HiScript^®^ II One Step RT-qPCR SYBR Green Kit was purchased fromVazyme Biotech (Nanjing, China). Duplex RT-qPCR-HRM was used to detect C-MDRV and Go-MDRV simultaneously. The 25 μL reaction mixtures contained 12.5 μL of 2× One Step SYBR Green Mix, 1.25 μL of One Step SYBR Green Enzyme Mix, 0.4 μL (10 μM) of each forward and reverse primer for C-MDRV, 0.4 μL (10 μM) of each forward and reverse primer for Go-MDRV, 1 μL of C-MDRV cDNA template, 1 μL of Go-MDRV cDNA template, and RNase-free H_2_O to make up final the volume of 25 μL. Amplification was performed on a LightCycler^®^ 96 Instrument using the following cycling program: 50℃ for 30 min, 95℃ for 30 sec, followed by 40 cycles of 95℃ for 5 s, 60℃ for 15 s and 72℃ for 6 s.

**2. Results**

The C-MDRV and Go-MDRV positive samples showed amplification curves, and single-peak melting curves with recorded *Tm* values of 82.5℃ and 85.0℃, respectively (Figure S2). Single-peak melting curves indicate the absence of primer dimers or non-specific amplification. The negative controls produced no CT values. The results indicated a 100% coincidence rate.


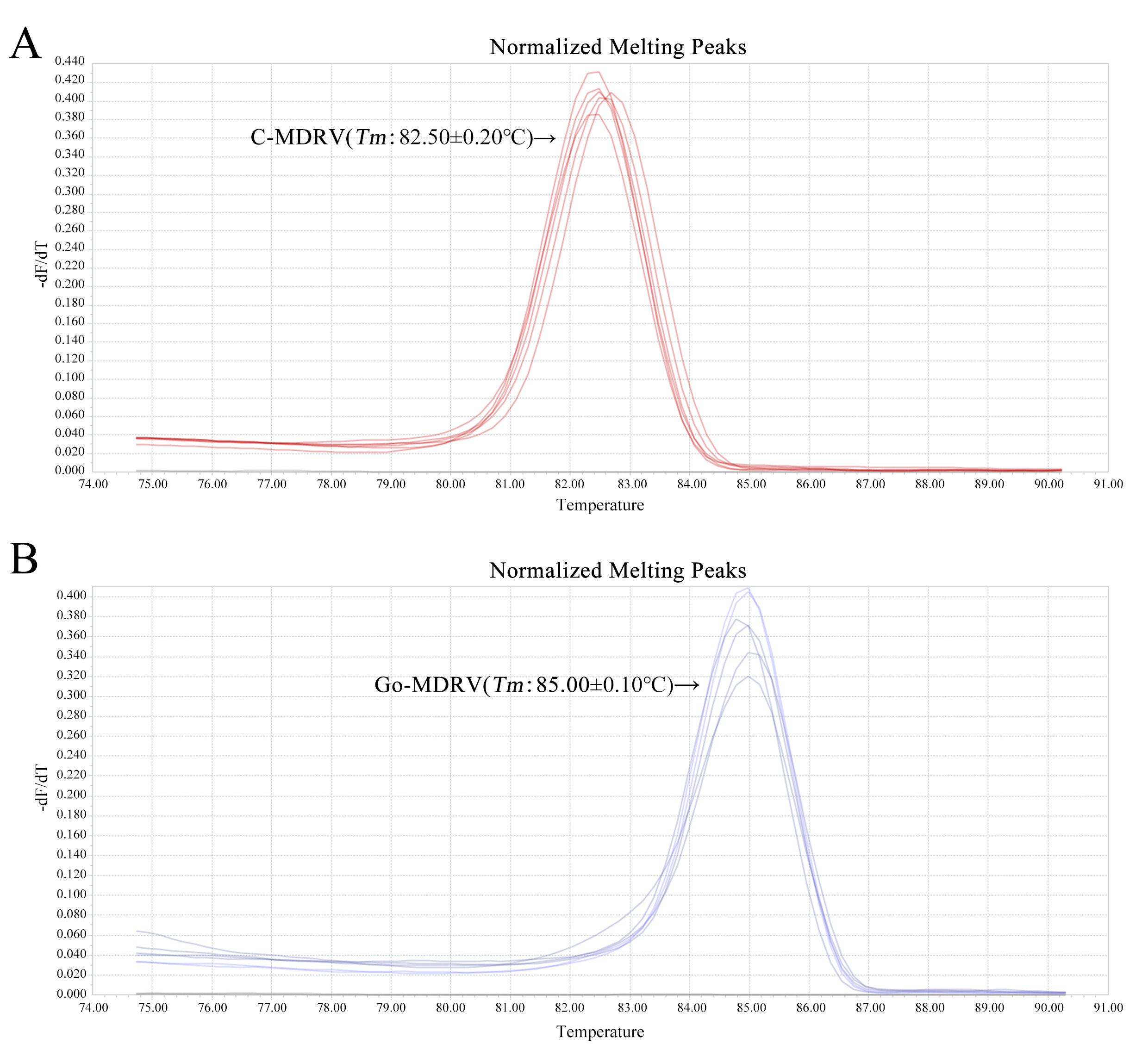


**FIGURE S2** C-MDRV and Go-MDRV detection were used one-step RT-qPCR-HRM and melting curve analysis. The *T_m_* values for C-MDRV and Go-MDRV were 82.5℃ and 85.0℃, respectively, indicateing that the one-step RT-qPCR-HRM detection method could also effectively distinguish C-MDRV and Go-MDRV.
